# Preventing Sudden Death And The Use Of Prophylactic Implanted Defibrillators

**Published:** 2003-04-01

**Authors:** Indranill Basu Ray

**Affiliations:** Cardiologist: Interventional Electrophysiology & Device Therapy, Arrhythmia Service, Department of Cardiology, St Michaels' Hospital, University of Toronto, Toronto, Ontario, Canada.

## Abstract

Implanted defibrillators have become mainstream therapy for the prevention of sudden cardiac death from ventricular tachyarrhythmias. A decade of studies has confirmed the superiority of ICDs over antiarrhythmic drug therapy in prolonging the life of patients with a prior history of sustained VT or VF.

More recent studies have compared ICD therapy to drugs or no antiarrhythmic therapy as 'primary prophylaxis' in patients considered at high risk for sudden death or with prior MIs. In selected patients, ICDs lead to important relative and absolute reductions in mortality in patients with no prior history of sustained VT or VF. Clinicians need to carefully consider these studies in their management of patients with CAD and severe LV dysfunction.

## Introduction

The understanding that cardiac death and, in particular, sudden cardiac death from fatal ventricular arrhythmias, is one of the most common causes of death in Western society is now widespread. Following the spectacular successes of thrombolytic, antiischemic, and revascularization therapies in the 1990's, focus has increasingly turned to the care of patients with the chronic consequences of coronary artery disease, chiefly left ventricular dysfunction and heart failure, and the propensity to sudden arrhythmic death. India being at present a nation facing an epidemic of coronary artery disease; would consequently have more & more of its populace in chronic CAD with risk of SCD.

Although it has been very clearly established that patients with left ventricular dysfunction, with or without symptomatic heart failure, are at high risk for out-of-hospital cardiac arrest, and undocumented but presumably arrhythmic sudden death, preventing such deaths has posed a major therapeutic challenge. First, it is difficult if not impossible to predict, with any reasonable degree of certainty, which particular patients are destined to suffer fatal arrhythmias, and which others are destined to remain clinically stable, or at least be free of serious ventricular arrhythmias. If one could identify such patients, then therapy could, of course, be targeted to only that select proportion whose destiny it is to suffer VT or VF (this represents approximately 40-60% of all patients with moderate to severe left ventricular dysfunction) [1]. Tests to identify patients at particularly high risk of sudden death have included the ability to induce ventricular tachycardia or fibrillation at invasive electrophysiologic study; the documentation of nonsustained ventricular tachycardia on holter or in-hospital ECG monitoring; the presence of subtle (not visible to the naked eye) ECG abnormalities of depolarization and repolarization using the filtered, signal-averaged ECG, or the presence of microvolt T-wave alternans; and the presence of abnormal autonomic modulation of cardiac function, by the registration of abnormally low heart rate variability (HRV), or depressed baroreceptor sensitivity.

Although each of these tests is of some prognostic value, they are insufficiently accurate, for practical clinical purposes, to direct therapy. Even amongst patients with poor ejection fraction, past history of myocardial infarction & the presence of ventricular scar are actually believed to be at high risk of sudden cardiac death. However there is no available diagnostic modality by which one can identify the exact population of patient liable to have SCD amongst thousands with these characteristics. This is tragic but true that this is in fact a serious limitation in our capability to triage patients requiring protection from SCD, as the incidence of sudden death amongst all patients with prior myocardial infarction is relatively low.

A second conceptual and practical problem is our inability to identify the timing, or proximate causes of sudden death from VT/VF. Such events appear to occur "out of the blue", and there are no clearly identifiable factors, which precede sudden cardiac death in most individuals. Although coronary artery disease is the most important etiologic factor leading to life threatening ventricular arrhythmias, angina, other manifestations of myocardial ischemia, sudden worsening of heart failure, or behavioural factors such as stress or exercise are rarely observed to immediately precede sudden death.

Improved acute and long term therapies have increased survival for patients with myocardial infarction, leading to a relative increase in the number of the patients with chronic coronary disease and left ventricular dysfunction, who are nevertheless stable and not expected to suffer imminent recurrent infarction or progressive heart failure. Such patients usually feel relatively well, and may require some persuasion to consider prophylactic therapy for cardiac arrhythmias, which from a subjective standpoint, can only decrease their quality of life in the short term. In confronting these dilemmas, clinicians through the 1980's and 1990's were optimistic that sudden death could be prevented by the administration of antiarrhythmic drug therapy. This approach had the conceptual benefit of being able to be delivered to a large group of patients at relatively low risk, as a "chemoprophylaxis" of sudden cardiac death. With the spectacular failure of class I drugs, for example with Flecainide following myocardial infarction, attention has turned to drugs that prolong cardiac repolarization (class III drugs). The most extensively studied of these drugs is amiodarone. Several very large trials have examined in detail the potential usefulness of amiodarone in preventing sudden death in high risk patients with coronary artery disease and left ventricular dysfunction, the largest being the EMIAT study (European Myocardial Infarction Arrhythmia Trial) [2], and the CHF-STAT study (Congestive Heart Failure - Survival Trial of Antiarrhythmic Therapy) [3]. The CAMIAT study (Canadian Amiodarone Myocardial Infarction Arrhythmia Trial) [4] also included patients with prior myocardial infarction, most of whom had at least moderate left ventricular dysfunction, as well as frequent ventricular premature beats. All of these studies were randomized placebo-controlled clinical trials. None were able to show neither a statistically significant, nor a clinically meaningful reduction in all cause cardiac mortality. Although meta analyses of amiodarone have suggested a small, and statistically significant reduction in all cause mortality in high risk populations, individual trials in patients with coronary artery disease and left ventricular dysfunction leaves us scant hope that amiodarone will be highly useful in this population of patients. The progressively increasing burden of adverse effects from amiodarone is another barrier to its use. Given the paucity of evidence, there is no good reason to prescribe amiodarone as primary prophylaxis for ventricular tachycardia or fibrillation in patients with coronary artery disease and left ventricular dysfunction, but no symptoms of sustained ventricular arrhythmias.

Large studies have also examined the potential benefit from new class III antiarrhythmic drugs in the prevention of sudden death following MI or with heart failure, including studies of dofetilide (DIAMOND [5] and DIAMOND-CHF [6]), and azimilide (the ALIVE study [7]). These also failed to show any difference between drug and placebo treated patients in sudden or all cause mortality. It is extremely important to note that beta-blocker therapy is of undoubted benefit in prolonging life in patients following myocardial infarction, particularly those with heart failure or extensive left ventricular dysfunction. Beyond the universal requirement for beta blockers unless absolutely contraindicated, there is however not much room for optimism that antiarrhythmic drugs, at least for the time being, will be even a partial solution to the problem of sudden cardiac death in susceptible coronary populations.

## The Implanted Defibrillator

The implanted defibrillator represents an effective, if intellectually inelegant therapy to prevent death from ventricular arrhythmias. The device, after all, does not prevent such arrhythmias but only treats them after they occur. Shocks are painful and unpleasant, the devices are expensive, a surgical procedure is required for its implantation, and the follow-up of patients can be technically challenging. Where do we stand with respect to the evidence concerning implanted defibrillators and sudden death?

There is extensive information regarding the *efficacy* of implanted defibrillator therapy. Appropriately tested devices have a 99% or greater probability of successfully restoring a perfusing rhythm in patients with ventricular tachycardia or ventricular fibrillation. Current devices can be implanted with a less than 1% major morbidity or mortality, with a surgical complexity and morbidity very similar to that of pacemaker implantation.

Studies in patients with a prior history of cardiac arrest, or sustained ventricular tachycardia; what is called the secondary prevention, have demonstrated convincingly that the implanted defibrillator is both effective, and superior to antiarrhythmic drug therapy in preventing all cause mortality in such patients. The AVID [8], CIDS [9], and CASH [10] studies, and their meta-analysis [11], have shown an approximately 20-30% reduction in all cause mortality in such patients. The greatest relative benefit from defibrillators over antiarrhythmic therapy (primarily amiodarone) occurs in those with the worst left ventricular function, and the elderly [12]. Since the majority of sudden deaths occur in patients without a prior history of documented sustained ventricular tachycardia or ventricular fibrillation, studies have assessed the usefulness of defibrillators as "primary prophylaxis" of sudden cardiac death. The accumulated evidence from these studies is briefly reviewed below.

## Clinical Trials

Initial trials focused on the selection of patients expected to be at particularly high risk of sudden cardiac death, based on a combination of low ejection fraction, and an additional risk marker for sudden cardiac death.

The MADIT I study assessed patients with coronary artery disease, poor left ventricular function, and asymptomatic nonsustained ventricular tachycardia, with inducible VT or VF at electrophysiologic study, not suppressible by antiarrhythmic drug therapy [13]. This study, the first to document a potential benefit from prophylactic ICDs, showed a 54% reduction in mortality in patients implanted with a defibrillator as opposed to those receiving "conventional medical therapy". The weaknesses of this trial included its relatively small size, inadequate therapy with beta blockers and ACE inhibitors, and the clinically impractical sequence of EP study and need for VT induction, followed by attempted VT/VF suppression with procainamide, that was required for risk stratification. Nevertheless, the results from this study led to FDA approval of implanted defibrillators for the particular subset of patients meeting the inclusion criteria for this study.

The CABG PATCH study randomized patients immediately following successful aortocoronary bypass surgery, if they met the inclusion criteria of a low ejection fraction (<35%), and a positive signal-averaged ECG, to either an implanted defibrillator or control therapy without the ICD [14]. All devices were attached to the heart by means of epicardial defibrillator patches (which are no longer used during the CABG procedure.

This study failed to show any benefit whatsoever from the implanted defibrillator, but both defibrillator and no defibrillator patients had a low cardiac mortality (5.9% per year), suggesting that surgical revascularization has a very important protective effect against sudden death.

The MUSTT study, like the MADIT study, also selected patients with coronary artery disease and ejection fraction of <40% if they had asymptomatic nonsustained VT on holter or in-hospital ECG monitoring, as well as inducible VT at EP study [15]. They were randomized to either "electrophysiologically guided", or no antiarrhythmic drug therapy. The electrophysiologically guided arm could include antiarrhythmic drugs designed to suppress the inducibility of ventricular tachycardia, those that would render inducible arrhythmias hemodynamically stable, or an implanted defibrillator. The choice between defibrillator versus drug therapy was not randomized.

Freedom at 5 years from sudden death was significantly lower in the electrophysiologically guided arm (25 vs. 32%, p=0.04) but all cause mortality was not (48 vs. 42%, p=0.06). However, a secondary analysis comparing patients with no antiarrhythmic therapy, the implanted defibrillator, and antiarrhythmic drug therapy, showed some striking trends. The relative risk of death from all causes in the ICD group compared to the no antiarrhythmic therapy group was 0.45 (95%, CI 0.32-0.63) and compared to electrophysiologically guided antiarrhythmic drug therapy was 0.40 (0.27-0.59). Although this is not strictly a randomized therapy assignment outcome, the study was widely and reasonably interpreted as showing superiority of the implanted defibrillator to no antiarrhythmic therapy or antiarrhythmic drug therapy. The observation that the "electrophysiologically guided" strategy was increasingly superior over no antiarrhythmic therapy as ICDs were increasingly frequently used over time, and relatively better in those centers that used ICDs more frequently, lent plausibility to the belief that it was the defibrillator which contributed all of the observed benefit of the antiarrhythmic therapy arm.

As a consequence of the MUSTT study, most expert bodies, stipulating guidelines for the treatment of ventricular arrhythmias, concluded that patients with coronary artery disease, ejection fraction <40%, and nonsustained VT, if they had inducible ventricular tachycardia at EP study, should preferably be treated with an implanted defibrillator [16].

Up until very recently, the database above was sufficiently ambiguous and related to a sufficiently select subgroup of patients (those with all of CAD, low ejection fraction, nonsustained VT, and inducible VT/VF at EP studies), that these recommendations have not been widely adopted in everyday clinical practice.

The MADIT II study, published in March 2002 [17], took a simplified approach to the testing of the hypothesis that implanted defibrillators would reduce all cause mortality in at risk populations. The only criteria to identify patients at risk from sudden death were the presence of coronary artery disease, a prior myocardial infarct, and an ejection fraction of <30%. This study randomized a total of 1232 patients to either the ICD (742 patients), or conventional medical therapy (490 patients, a 3:2 ratio). Neither nonsustained VT nor an electrophysiologic study was required for entry into this study.

The patient population in this study was reasonably representative of a potentially very large group of patients with chronic coronary artery disease and prior MI. The mean age was 65 years, and 70% of patients were either NYHA class II or I. A majority had a remote history of coronary bypass surgery (57%), or coronary angioplasty (44%). In the vast majority, more than 6 months had elapsed since their most recent MI. Interestingly enough the associated drug therapy that most patients in the trial had was sufficiently appropriate as to allow generalizability in this trial. Seventy percent were receiving ACE inhibitors, 70% beta-blockers, and 57% digitalis. Sixty-six percent received statins. About 12% were receiving amiodarone at last contact (presumably most often for atrial fibrillation), and only 9% received calcium channel blockers and 3% received class I antiarrhythmic drugs.

Patients were followed to a common primary endpoint of death from any cause. The pre-specified mortality efficacy boundary was achieved just over 4 years after the study began, after an average follow-up of 20 months.

The defibrillator therapy resulted in an increasing mortality benefit over conventional therapy over time, with an aggregate 31% reduction in the risk of death at any time interval, including a relative decrease of mortality of 12%, 28%, and 28% at 1, 2, and 3 years respectively. In absolute terms, this meant a 1%, 6%, and 9% reduction in mortality at 1, 2, and 3 years; in other words, the number needed to treat (NNT) to prevent 1 death by 3 years was approximately 11. This NNT compares very favorably to other cardiovascular therapies in common use, for example beta-blockers (CIBIS 2, NNT = 23), statins (4S, NNT = 28), or ACE inhibitors (SAVE, NNT = 20). There was a slightly higher probability of hospitalization for heart failure in the ICD group (11 per 1000 months), versus the control group (9 per 1000 months, p=0.09).

Subsequent further subgroup analysis showed that patients with QRS prolongation of >120 msec at baseline received a particularly and dramatically large benefit from the implantation of an ICD, the mortality reduction being from 53% to 21% (a 63% reduction) at 3 years in these patients. This latter observation is consistent with prior demonstration of QRS prolongation on the surface ECG as being particularly potent, simple marker for the probability of all cause mortality and sudden death.

## Present Status of Prophylactic ICDs

The evidence indicating that implanted defibrillators prolong life in patients who are susceptible to sudden cardiac death is compelling. It is important to underline that the trials pertain exclusively to patients with coronary disease (as opposed to those with dilated or other forms of cardiomyopathy), and probably are not applicable to patients immediately after bypass surgery. No study has shown superiority of ICDs over medical therapy in patients with dilated cardiomyopathy, and a large study of ICDs vs. amiodarone (SCD-HeFT) will address this question. With these exceptions, such patients with very poor ventricular function unquestionably benefit from the implantation of a defibrillator, even if they are receiving optimal medical therapy. Although the MADIT and MUSTT trials did not systematically compare the ICD to "best" medical therapy (almost certainly amiodarone), the absence of clear proof that amiodarone is effective, and the toxicity burden from amiodarone (which itself increases progressively over time), suggests that for the moment the defibrillator should be considered clearly superior to amiodarone therapy or no antiarrhythmic therapy in the prevention of sudden and all cause mortality in susceptible populations. Importantly, defibrillators in the MADIT study and other studies were implanted with virtually no perioperative mortality, and a 2.5% incidence of non-fatal adverse events requiring surgical interventions (lead problems or infection) [17]. The main barrier to more widespread use of prophylactic implanted defibrillators, at least in the Indian context; though it applies as well to the west particularly in countries with government funded health care system, seems to be resource limitations, both with respect to device and implantation costs, and the availability of medical personnel to perform the procedures and follow the patients. In addition, the total number of years added to life, as well as the quality of these added years, is not fully elucidated given the relatively short follow-up time of all of the studies published thus far.

## Who is The Appropriate Candidate?

For the time being, it seems appropriate to at least *consider* a prophylactic implanted defibrillator in all patients with a history of remote myocardial infarction and ejection fraction of <30%, provided they are receiving or have been considered for evidence based pharmacological therapies including beta-blockers, ACE inhibitors, aspirin, statins, and spironolactone as indicated. If revascularization is indicated and feasible, it should be performed. The presence of nonsustained ventricular tachycardia on in-hospital or Holter monitoring probably adds some prognostic significance, although the amount of information contained in this finding is not clear. Performing an electrophysiologic study for risk stratification is probably not required for most such patients. The expectation of treatment benefit is amplified in patients with bundle branch block or QRS >120 msec.

Following these considerations, it is appropriate and should be considered advisable to at least inform the patient of the treatment options available, unless there are severe co-morbidities, which reduce the expectation of treatment benefit.
